# Prediction of COVID-19 Using Genetic Deep Learning Convolutional Neural Network (GDCNN)

**DOI:** 10.1109/ACCESS.2020.3025164

**Published:** 2020-09-21

**Authors:** R. G. Babukarthik, V. Ananth Krishna Adiga, G. Sambasivam, D. Chandramohan, J. Amudhavel

**Affiliations:** 1 Department of Computer Science and EngineeringDayananda Sagar University 508654 Bengaluru 560078 India; 2 Faculty of Information and Communication TechnologyISBAT University Kampala Uganda; 3 Department of Computer Science and EngineeringMadanapalle Institute of Technology and Science 210883 Madanapalle 517325 India; 4 School of Computer Science and EngineeringVIT Bhopal University Bhopal 466114 India

**Keywords:** Genetic Deep Learning Convolutional Neural Network (GDCNN), Computed Tomography (CT), Chest X-Ray (CXR), Artificial Intelligence (AI)

## Abstract

Rapid spread of Coronavirus disease COVID-19 leads to severe pneumonia and it is estimated to create a high impact on the healthcare system. An urgent need for early diagnosis is required for precise treatment, which in turn reduces the pressure in the health care system. Some of the standard image diagnosis available is Computed Tomography (CT) scan and Chest X-Ray (CXR). Even though a CT scan is considered a gold standard in diagnosis, CXR is most widely used due to widespread, faster, and cheaper. This study aims to provide a solution for identifying pneumonia due to COVID-19 and healthy lungs (normal person) using CXR images. One of the remarkable methods used for extracting a high dimensional feature from medical images is the Deep learning method. In this research, the state-of-the-art techniques used is Genetic Deep Learning Convolutional Neural Network (GDCNN). It is trained from the scratch for extracting features for classifying them between COVID-19 and normal images. A dataset consisting of more than 5000 CXR image samples is used for classifying pneumonia, normal and other pneumonia diseases. Training a GDCNN from scratch proves that, the proposed method performs better compared to other transfer learning techniques. Classification accuracy of 98.84%, the precision of 93%, the sensitivity of 100%, and specificity of 97.0% in COVID-19 prediction is achieved. Top classification accuracy obtained in this research reveals the best nominal rate in the identification of COVID-19 disease prediction in an unbalanced environment. The novel model proposed for classification proves to be better than the existing models such as ReseNet18, ReseNet50, Squeezenet, DenseNet-121, and Visual Geometry Group (VGG16).

## I. Introduction

Novel coronavirus has been formally named as Severe Acute Respiratory Syndrome Coronavirus-2 (SARS-COV-2) is responsible for causing Coronavirus Disease 2019 (COVID-19) [1]. Few symptoms of COVID-19 are cough, fever, a disease of the respiratory system and in some cases, it leads to pneumonia [2]. Generally, pneumonia is termed as the infection that causes inflammation to air sacs present in the lungs for oxygen transfer. The other way of pneumonia infection is fungi, bacteria, and other viruses. The reason for severity is chronic diseases such as bronchitis or asthma, impaired or weak immune system, smoking, and aging people. The infected peoples are treated based on the infected organism, however, cough medicine, pain reliever, fever reducer, and antibiotics are given to patients based on the symptoms. If the patient is severely affected, they have to be hospitalized and treatment must be given in the Intensive Care Unit (ICU), if needed ventilator to be provided for breathing [3]. The pandemic of COVID-19 is due to its seriousness and its faster transmissibility [4]. Greater impact in the health care department is mainly due to the number of people getting affected day by day, as they need to provide mechanical ventilator for the serious patient admitted in ICU. Hence, number of beds in ICU also need to be increased drastically [5]. In the above situation, the initial diagnosis is vital for proper treatment which, in turn, reduces the pressure on the health care system.

Artificial Intelligence (AI) provides a major breakthrough for the diagnosis of COVID-19 and other types of pneumonia. Pneumonia is diagnosed using some of the standard images such as Computed Tomography (CT) scan and Chest X-Ray (CXR). The primary source for evaluating pneumonia is CXR, as CXR leads to misdiagnosis and less precision. However, CXR is used because of its cheaper rate, less exposure to radiation for patients, faster, and it’s readily available in all health care systems [6]. Identification of pneumonia is no easy task, as the reviewer needs to look into the white patches present in the lungs and most of the air sacs filled with water or pus hence, it is a tedious process to differentiate between bronchitis or tuberculosis [7].

The concepts of COVID-19 pandemic and pneumonia disease, hierarchical, and flat classification merge in our work are presented in this section. Furthermore, background related to data imbalances is also discussed.

### A. COVID-19 Pandemic and Pneumonia Disease

The first COVID-19 case was reported in Wuhan, China, and it is gradually starting to spread across the rest of the world within a short interval of time. This indicates that the number of cases reported increases exponentially, as of now more than 8.24 million confirmed cases worldwide [1]. COVID-19 Epidemiological characteristics are still under the process of investigation, evidence prove that more or less, 80% of patients are in mild condition with few asymptomatic and approximately 20% are in severe condition among, this 10% have to be in ICU with ventilators [8]. The most important concern is the number of patients admitted to the ICU as there are only limited beds. The major problem of COVID-19 is pneumonia, as it infects a portion of the lung, which transfers gas termed as pulmonary parenchyma. Some of the organisms like fungi or bacteria and viruses are also present. Generally, pneumonia is termed as a group of diseases, hence diagnosis also needs to be different, therefore, Chest X-Ray image and CT scan used for diagnosis [9].

### B. Classification of Classes

Flat classification involves multi-label, binary, and multi-class classification problems, however, multi-label includes multiple classes, and the output is associated with each other. Binary classification is stated as the task of classifying the images from the given dataset into two categories on the basis of classification rules. Some of the methods used for classification are random forests, decision trees, support vector machines, Bayesian networks, probit models, neural networks, and logistic regression. Table 1 shows the parameters explanation in terms of symbol and its explanation. The features are represented by ‘x’ containing a set of parameter”$x_{1,} x_{2} $‘’ and it is shown in equation (1). The output is represented by ‘y’ as in equation (2), a decision function based on the weight for each parameter is evaluated using equation (3). The algorithm function is represented by equation (4), thus the number of parameters is based on the real value function using equation (6).

\begin{align*}&{\mathrm {Features}}~ x=(x_{1},x_{2})\tag{1}\\&{\mathrm {Target}} ~(y\in \{-1,1\})\tag{2}\end{align*}

Decision function

\begin{equation*} d(x)=w_{0} +w_{1} x_{1} +w_{2} x_{2}\tag{3}\end{equation*}

Algorithm function

\begin{align*} a({\mathrm {x}})=&{\mathrm {sign}}({\mathrm {w}}^{T}x) \tag{4}\\ d({\mathrm {x}})>&0\tag{5}\end{align*}

Number of Parameter

\begin{equation*} {\mathrm {d}}(w\in {\mathrm{ R}}^{d})\tag{6}\end{equation*}

It is clear from the above context that pneumonia is based on multi-class classification, as many features need to extract from CXR images however, one label needs to be associated. Silla *et al.* stated taxonomy is considered to organize a tree hierarchy defined on the basis of incomplete order set [10] Moreover, the various ways to handle hierarchical classification problems in regards to labeling classification process also discussed [11]. Local classifiers (LC) are an approach that considers hierarchy and partial local information perception and thus allowing the multi-class/binary classifiers to handle the problem in a local manner [12], [13]. Furthermore, the Global Classifier (GC) approach is a unique classification model built based on the training dataset. Considering class hierarchal as a whole, thus significant information on the pneumonia labels is found in the entire class hierarchy, thus, GC approached is widely used [14], [15].

**TABLE 1:** Parameter Explanation

Sl.No.	Symbol	Explanation
1.	x	Parameter features
2.	y	Output
3.	w	Weight
4.	d(x)	Decision function
5.	a(x)	Algorithm function
6.	Sign(x)	Sign function
7.	R	Number of parameter
8.	k	Number of classes
9.	z	Linear model
10.	$\sigma (z)$	Softmax transformation
11.	p	Class probabilities
12.	${\mathrm {S}}_{f} $	Size of the filter
13.	${\mathrm {N}}_{f} $	Number of filter
14.	${\mathrm {B}}_{n} $	Batch normalization
15.	P	Pooling
16.	D	Dropout
17.	A	Activation
18.	O	Optimizer
19.	N	Convolutional block
20.	$P_{ODV_{i}} $	Ordered Distance Vector population
21.	$c_{l} $	Code length
22.	$\odot$	Individual population
23.	o	Total number of individual
24.	n	Size of problem instance
25.	TP	True Positive
26.	TN	True Negative
27.	FP	False Positive
28.	FN	False Negative

Multi-class classification output is given by ‘y’ using equation (7), the number of parameters is stated by the equation (9) and classification accuracy using equation (10).

Multi class classification

\begin{align*}&(y\in \{1\ldots \ldots {\mathrm {k}}\}) \tag{7}\\&a(x)=\arg \_{}\max (w_{k}^{T} x).{\mathrm {k}}\in \{1\ldots.{\mathrm {k}}\}\tag{8}\end{align*}

Number of parameter

\begin{equation*} k\ast d\{{\mathrm {w}}_{k} \in {\mathrm {R}}^{d}\}\tag{9}\end{equation*}

Classification accuracy

\begin{equation*} \frac {1}{l}\sum \limits _{i=1}^{l} {[{\mathrm {a}}({\mathrm {x}}_{i})=y_{i}]}\tag{10}\end{equation*}

Class probabilities and the class score based on logits from a linear models using equation (11)

\begin{align*}&z=({\mathrm {w}}^{T}{\mathrm {x}}_{1},\ldots {\mathrm {w}}_{k}^{T} {\mathrm {x}}) \tag{11}\\&\quad ({\mathrm {e}}^{z_{1}}\ldots \ldots.{\mathrm {e}}^{z_{k}})\tag{12}\end{align*}

Applying Softmax transform is represented by equation (13)

\begin{equation*} \sigma ({\mathrm {z}})=\left({\frac {e^{z_{1}}}{\sum \limits _{k=1}^{k} {e^{z_{k}}} }\ldots \ldots..\frac {e^{z_{k}}}{\sum \limits _{k=1}^{k} {e^{z_{k}}}}}\right)\tag{13}\end{equation*}

Loss function

Multi class loss function is predicted by class probabilities model output using equation (14)

\begin{equation*} L_{s} =-\frac {1}{M}\sum \limits _{i=1}^{M} {\log } \frac {\exp ({\mathrm {w}}_{yi}^{T} {\mathrm {f}}({\mathrm {x}}_{i})+{\mathrm {b}}_{yi})}{\sum \limits _{j=1}^{C} {\exp ({\mathrm {w}}_{j}^{T} {\mathrm {f}}({\mathrm {x}}_{i} +{\mathrm {b}}_{j}))} }\tag{14}\end{equation*}

M = mini batch size, $f(x_{i}) =$ corresponding output of the penultimate layer of the DCNN, C = number of classes, w = last layer weight and b = last layer bias.

Target value for class probabilities using equation (15)

\begin{equation*} p=([y=1],[y=k])\tag{15}\end{equation*}

Similarity between ‘z’ and ‘p’ can be measured by the cross entropy using equation (16)

\begin{equation*} -\sum \limits _{k=1}^{k} {[{\mathrm {y=k}}]\log \frac {e^{z_{k}}}{\sum \limits _{j=1}^{k} {e^{z_{j}}}}} =-\log \frac {e^{z_{y}}}{\sum \limits _{j=1}^{k} {e^{z_{j}}}}\tag{16}\end{equation*}

### C. Imbalanceness Data and Resampling

Class imbalance distribution problems have been faced by most researchers whenever, they deal with the real datasets [16], [17]. However, classifier focus on minimizing the global error rate, thus the algorithm concentrates on the majority classes, but, it also focuses on minority classes based on the problem domain such as medical image classification and credit card fraud detection [18], [19]. In the real world, classifying pneumonia type using CXR images is also considered as imbalanced learning as there are only a few people with affected pneumonia than considering health persons [20], [21]. On the other hand, the number of people affected by various types of pneumonia disease is also imbalance [22], [23]. Currently, the number of people affected by COVID-19 is very larger than compared with the people affected by MERS, Severe Acute Respiratory Syndrome Coronavirus (SARS), Streptococcus, Varicella, and Pneumocystis [24], [25]. The imbalance problem in classification datasets has been resolved by the various authors and one such technique is data level solutions [26], [27]. The techniques focus on re-balancing the class distribution using resampling the dataset which reduces the consequence of class imbalances, in other words, before the training phase, pre-processing the dataset has to be done [28], [29]. Resampling further subdivided into two types, undersampling and oversampling. Both types are used to fine-tune the class distribution of a dataset, thus, it is the ratio among the various classes in the datasets. Undersampling includes removal of majority classes for distribution of samples, whereas, in oversampling few instances are duplicated to main the class distribution [30].

### D. Deep Convolutional Neural Networks (DCNNS)

The standard of many computer version tasks is greatly enhanced over the period with the help of Deep convolutional neural networks (DCNNs), some of them are GoogLeNet [31], AlexNet [32], DenseNet [33], VGGNet [34] and ResNet [35]. DCNN is a professional system that reduced the infinite amount of human expertise involved in the analysis of data. Thereby, providing an identical feature extraction classification model to reduce the burden of handcrafted extraction in case of network design. In designing DCNN architecture, the main ambition of artificial intelligence is to develop an autonomous learning system with less human intervention also needs to consider [36]. In recent years research focused more attention in automated design of DCNN architectures, which in turn leads to the development of many algorithms and it is generally categories into four groups: (1) Evolutionary optimization of DCNN architectures, (2) Optimization in DCNN architectures using deep learning, (3) DCNN architectures, selection in an available group of candidates and (4) DCNN architecture optimization using reinforcement learning. Among the four categories, evolutionary optimization proved to be a more promising approach in the case of multi-point global search thereby, leading to high-quality optimization solutions in complex search space [37]. Despite the success, many evolutionary methods have some restrictions on DCNN architecture some of them are fixed filter size, fixed pooling size, fixed depth, avoiding pooling operation [38], or avoiding crossover operation [39] and fixed activation function [40]. All these restrictions leads to a reduction in computational complexity which in turn leads to performance degradation. Thus, for parallel optimization using evolutionary methods, thousands of computers are required [41].

Many classifiers that incorporate Genetic Algorithm (GA) techniques use a single–phased GA such as Non-dominated sorting GA-II (NSGA-II) (Deb *et al.* 2002). A classifier model based on bi-phase needs to be developed which includes classification rule extraction. Local heuristic search techniques are used for pattern discovery with rule induction methods in case of data mining (Chiu *et al.* in 2005). The major issue with the local search method is that it frequently gets trapped with local optima and furthermore it is sensitive to the initial solution. The above drawback is overcome by using GA as it discovers the best classification rules, moreover, local optimum issued is also addressed. Better feature interaction is done with GA than compare to the greedy rule induction algorithm, (Freitas *et al.* in 2003) some of the issues that exist in the GA is that, as there is no guarantee of achieving global optimum and its computational cost linearly increases with the search space. Fuzzy classification rules are easily understood by humans as it deals with the uncertainty problems but the only drawback is that the complexity involved is more in fuzzy classification rule extraction than crisp classification rule extraction (De Jong *et al.* in 1988).

In this research, the proposed genetic-based DCNN design for spontaneously producing the architecture of a DCNN to solve image classification issues. The complexity involved DCNN architecture is reduced by developing a suitable encoding scheme which includes all the operations performed in the DCNN, some of them are pooling convolution, activation, batch normalization, drop out, optimizer, and full connection is encoded in the form of integer vectors.

The main aim of this research is to explore the various types of pneumonia due to pathogens using CXR images. CXR image samples are used because of its advantages in terms of faster and minimal cost. Even though CT scans hold the better standard in the diagnosis of pneumonia, the major setback is it is costly and scarce. Our main aim is to predict COVID-19 pneumonia using CXR images, as it is widely spread across the world. The validation of the precision is done using a Macro-Avg F1-score. Since the database is imbalanced known resampling techniques are used. CXR images are analyzed by identifying the texture which is one of the main attributes present, thus, exploring a few texture descriptors used for training CNN models. A hierarchical classification approach is used for extraction features and a Genetic based deep learning approach is used for prediction of COVID-19 and other pulmonary diseases. Dataset consisting of more than 5000 image samples is chosen for prediction of COVID-19, from publicly available dataset consisting of 2, 24,316 Chest X-ray.

Section I describes the pandemic situation in the health care system due to COVID-19 and the early reasons for diagnosing it. Section II states the existing techniques used for classification of images and Deep Learning Convolutional Neural Network models used for prediction of COVID-19. Section III depicts the proposed Genetic Deep Learning Convolutional Neural Network comprising of Ordered Distance Vector population techniques for optimal prediction of COVID-19. Section IV represents the experimental analysis of the proposed work and it is compared with the existing DCNN models. Some of the parameters used for performance analysis are sensitivity, accuracy, specificity, recall, precision and F1-score. Final conclusion of the proposed GDCNN models in future works.

## II. Related Works

In-depth study of various techniques used for classification of images is performed. Furthermore, discussed the existing DCNN models used for prediction of COVID-19 using CT and CXR images. The analysis is stated in terms of accuracy for various prediction models. Comprehensive study is performed in automation of DCNN architecture for searching and classification of images.

Nanni *et al.* in 2010 [41] compared different texture descriptors which are handcrafted and obtain from Local Binary Pattern (LBP) used in medical applications. Three different LBP evaluators are Elongated Quinary Pattern (EQP), Local Ternary Pattern (EQP), and Elliptical Binary Pattern (EBP) [42]. These descriptors are calculated on various medical applications such as classification of cell phenotype image with 2D- hela dataset [43]. Detection of pain expression with a facial image of COPE database and Papanicolaou test used for diagnosing cervical cancer. Data is collected from Herlev university containing 917 images obtained from the microscope and digital camera. Support Vector Machine (SVM) is used for validating the EQP descriptor and it performs comparatively better for all the tasks. Parveen *et al.* in 2010 [44] carried a texture analysis of images used in radiotherapy applications. The mathematical technique stating grey-level patterns in case of tumor heterogeneity. Specially focused on tissue, causing radiation and for tumor, analysis is performed based on radiotherapy medical images. The major drawback of this technique is that it lacks in the biological interpretation of predicting tissue infected by radiation [45].

Zhou *et al.* in 2020 [46] suggested a deep learning model for distinguishing influenza pneumonia taken from CT images and novel coronavirus pneumonia. CT images are better than CXR images as it shows pulmonary infection clearly but it’s much costlier. Li *et al.* [47] identified COVID-19 using Artificial Intelligence (AI), thus dataset comprising of affected COVID-19 images, various pneumonia, and diagnosed patients with pneumonia. The images are gathered from Chinese hospitals containing 2969 images of the training set, viral pneumonia 1396, more than 400 images of COVID-19 patients, and 1173 non-pneumonia. K. He, X. Zhang *et al.* in 2016 [34] stated the 3D learning model for prediction of COVID-19, non-pneumonia, and various viral pneumonia and CT image is given as input. The output of the prediction clearly shows that for COVID-19 AUROC value is 0.96 and for other viral pneumonia is 0.95. Narin *et al.* in 2020 [48] detected COVID-19 using CXR images with three unique deep neural networks such as InceptionResNetV2, ResNet50, and Inception-V3. The dataset consists of 100 CXR images comprising of 50 COVID-19 positives and 50 COVID-19 negatives. The result is validated using a fivefold cross, where 87% of accuracy is achieved for inception-ResNetV2, 97% for Inception-V3, and 98% using the ResNet50 model. Gozes *et al.* in 2020 [49] detected the COVID-19 using deep learning models with CT images as an input. The evolution is performed for patients with the help of 3D volume, thereby, producing cornea score. The main aim of the work is to track the progress COVID-19, the dataset consists of 157 CT images collected from the USA and China. Furthermore, detection has been carried out using 3D and 2D deep learning models, with few changes in the already existing AI models and associated with clinical understanding. With AUROC of 0.996 differentiating with non-corona image and cornea images.

Wang *et al.* in 2020 [50] developed COVID-Net which is an open-source deep neural network used for detecting COVID-19 with CXR images. The dataset is created in such a way it supports COVID-Net experimentation thus, comprising of 16,756 patients. COVID-Net architecture developed on the basis of best practices and human-driven design merged with network architecture. The detection is performed with 92.4% accuracy, sensitivity rate 95%, and the infection rate is 80%. Khan *et al.* in 2020 [51] developed a CoroNet using Convolutional Neural Network (CNN) for detecting COVID-19 with input as CXR images, moreover, this model is based on Extreme Inception which consists of 71 layers of trained images using ImageNet dataset. It detects that 330 patients are affected by bacteria, 310 normal patients, 284 COVID-19, and 327 viral. F1-scores of 0.93 and 0.87 average accuracies, the major problem with this approach is the dataset used, as it is not publicly available. Furthermore, the hierarchical classification is not addressed. Ozturk *et al.* in 2020 [52] stated a deeper model for the detection of COVID-19 with CXR images, thus, classification is performed based on binary and multiclass. The proposed model obtains an accuracy of 98.08% in the case of binary classification, whereas, 87.02% for multi-class.

Many researchers contributed a lot of effort in automating DCNN architectures for searching and classification of images. Jin *et al.* [53] proposed super-modular and sub-modular optimization in the construction of DCNN architecture and proposed rules for setting the depth and width of DCNN. Fernando *et al.* [54] proposed a new algorithm, termed PathNet algorithm which models sub-network from super DCNN architecture and proven that the proposed algorithm is capable of supporting transfer learning both in reinforcement and supervised learning settings. Moreover, optimization of the architecture/weights of DCNN is done using another deep neural network. Ha *et al.* [55] used a Discrete Cosine Transform (DCT) and hyper network to progress weights of fixed DCNN architecture. De Barbandere *et al.* [56] used producing filters for DCNN architecture to take care of dynamic filter networks, which is divided into a dynamic filtering layer and filter-generating network. The filter generating network produces runtime sample-specific filter parameters based on input condition and dynamic filters use those filters as an input. Nowadays reinforcement learning is used in design architectures of DCNN, Zoph and Le [57] maximize the accuracy of image validation in DCNN architectures using the recurrent neural network, which is trained by reinforcement learning, however, in this technique, a fixed depth in DCNN architecture is created on each layer by layer, thus allowing a fixed number of filters and fixed filter size. Furthermore, it uses asynchronous parameters with 800 graphs processing units (GPUs) and distributed training.

Baker *et al.* in 2016 [58] on the basis of reinforcement learning introduced MetaONN techniques for DCNN architectures. Furthermore, the techniques use a Q-learning agent to exploit and explore the space ideal architectures based on experience replay and greedy strategy. In the design of the neural networks, many evolutionary algorithms have already been applied. Miikkulainen *et al.* in 2017 [59] proposed techniques containing all neurons associated with DNA, produced architecture by mutation techniques are divided into three types (1) weight modification (2) whenever splitting connection occurs a new neuron had to be inserted (3) a new connection is added to the existing connections. Suganuma *et al.* in 2017 [60] suggested the CoDeepNEAT algorithm where chromosomes populations are created with minimal complexity. Furthermore, the structure is added iteratively via mutation generations. Minaee *et al.* [61] proposed genetic programming for DCNN architecture and it is encoded by Cartesian genetic programming which is directed acyclic graphs having a two-dimensional grid-based on computational neurons moreover, it is said to be a more dominant algorithm. The algorithm uses a heuristic search for selection and fitness function. Wang *et al.* in 2020 [62] proposed Genetic DCNN based on a fixed-length binary string encoding scheme, only the pooling layer is considered for encoding and thus neglected fully connected layer thereby it leads to minimum number of layers with least filter number and filter size. Real *et al.* [40] designed a DCNN architecture for CIFAR-100 dataset with the help of GA, the DNA encoding scheme is used. Basically, this architecture originated based on the mutation operations furthermore, encounters filters size is dealt with by restructuring non-primary edges along with interpolation. DCNN architecture produced by these techniques is fully trained and uses a distributed algorithm which involves more than 250 computers. Desell *et al.* [39] proposed the EXACT method comprising flexible filter size and connection on the basis of GA using asynchronous evolutionary techniques. It includes more than 4500 dedicated computers. The MNIST dataset is used to train the model for 120000 DCNN [53].

Matteo Polsinelli *et al.* in 2020 [63] proposed a light Convolutional Neural Networks (CNN) for diagnosing COVID-19 using CT images. Few changes in the SqueezeNet CNN model are made, thereby, achieving 83% of accuracy, specificity of 81%, 81.73% of precision, F1-score of 0.8333 and with 85% of sensitivity. Shreshth Tuli *et al.* in 2020 [64] proposed Long-Short-Term-Memory method based on Weibull for predicting started and ending the cycle of COVID-19. Basically, this model is used for understanding the relationship between infection rate and deaths. The model works on cloud which is useful for dynamic prediction and helpful in providing guidelines for administration, policy makers and health care system. Adarsh Kumar *et al.* in 2020 [65] proposed a drone based on network system for identifying the number of people affected by COVID-19. The model is deployed in remote and congested areas, where there doesn’t exist internet or wireless connectivity. The model is used for health care system for sanitizing and identifying the infected patients. Parnian Afshar *et al.* in 2020 [66] proposed a framework based on capsule network termed as COVID-CAPS for identifying COVID-19 using X-ray images. The major drawback of the proposed method is that it is used for small datasets, however, accuracy of 95.7%, specificity of 95.8 and a sensitivity of 90% is achieved.

The existing models proposed by various author lacks in the accuracy and the computation time for prediction of COVID-19 is also considerably larger. Hence, there arises a need for early prediction. The architecture needs to be continuous and autonomous learning algorithm for early diagnosis using XRay image samples.

## III. Method

Proposed an independent and continuous learning algorithm for generating a DCNN architecture spontaneously. The process includes the operations of partitioning DCNN into numerous weighted fully connected and meta convolutional block. Each block possesses the operations like pooling, convolution, batch normalization, dropout, fully connection and activation operation. Thereby converting the DCNN architecture into a standard integer code. The genetic operations such as selection, crossover and mutation process are performed to evolve the population for DCNN architectures. The individual population is increasing and progressed using the design of the proposed genetic DCNN. Furthermore, encoding is performed with acceptable DCNN architecture. Population initialization is performed randomly using random function, moreover fitness of each individual is calculated based on the performance of genetic DCNN encoding used for specific image detection problems. On the basis of the existing generation, a new generation is performed using genetic operators such as selection, crossover operator, and mutation for improvising overall fitness values. The evolution is carried out in iteration manner based on generation- by- generation till it reaches the criteria or for a particular generation number.

### A. Encoding Scheme

The proposed genetic based DCNN architecture evolved on the basis of locus on a chromosome. Thus chromosomes are divided into two parts, namely, q-arm and p-arm. Gene map is termed as the method of loci known for a specific genome. Operations need to be performed by DCNN is observed as the loci on a chromosome, thereby, it is clear that, based on gene map all the encoding operation of DCNN is performed. Basically, convolutional block has five major operations, they are, convolution, pooling, normalization, drop out and activation. Furthermore, convolution operation contains two parameters such as the size of the filter ‘${\mathrm {S}}_{f} $’ and number of filter ‘${\mathrm {N}}_{f} $’. Convolutional block has 6 loci (${\mathrm {loci}}_{chrom} =6$) in the order as $[{\mathrm {N}}_{f} {\mathrm {S}}_{f} {\mathrm {B}}_{n} {\mathrm {PDA}}]$, ${\mathrm {B}}_{n} $ state batch normalization, ‘P’ for pooling, ‘D’ dropout and ‘A’ activation. Likewise, fully connected block has four loci in an orderly manner and encoded as $[{\mathrm {N}}_{f} {\mathrm {B}}_{n} {\mathrm {DA}}]$. The DCNN architecture is also influenced by the optimizer ‘O’.

Table 2 shows the range of values at every locus of the code, (${\mathrm {N}}_{f}$) various from 16 to 512, ${\mathrm {S}}_{f} $ are $7\times7$, $5\times 5$ and $3\times3$, pooling operation is indicated by three values they are 0, 1 and 2. ‘0’ denotes no pooling, ‘1’ state’s maximum pooling and ‘2’ for average pooling. Usually ${\mathrm {B}}_{n} $ take the value ‘1’ and ‘0’, ‘1’ indicates batch normalization is performed and ‘0’ not performed. ‘A’ various from 0 to 5 stating ELU [2], ReLU [8], PReLU [12], TReLU [18], softmax and LeakyReLU [22]. The value of ‘O’ ranges from 0 to 6 denoting SGD [6], Adadelta [17], Adamax [31], Adam [31], Adagrad [35] and RMSprop [36]. Thus, based on the coding scheme p-arm contains the sequence [$\text{N}_{\mathrm {f}}~\text{S}_{\mathrm {f}}~\text{B}_{\mathrm {n}}$ PDA] $[{\mathrm {N}}_{f} {\mathrm {S}}_{f} {\mathrm {B}}_{n}]$ and q-arm sequence is [$\text{N}_{\mathrm {f}}~\text{S}_{\mathrm {f}}~\text{B}_{\mathrm {n}}$ DA].

**TABLE 2:** Parameter Range

Sl.No.	Symbols	value
1.	${\mathrm {N}}_{f} $	[16,.....512]
2.	${\mathrm {S}}_{f} $	7,5,3
3.	${\mathrm {B}}_{n} $	0,1
4.	A	0,1,2,..4
5.	D	[0,0.5]
6.	P	0,1,2
7.	O	0,1,2,.....6

### B. Initialization

Ordered Distance Vector population initialization techniques are used, which inhabit individual diversity, randomness and potential sequence. This is shown using equation (17). The individual populations are produced and this type of population has a more potential permutation of images and better individual diversity. Thus, it is more effective and better solution with minimum convergence time.

\begin{align*} \boxed { P_{ODV} =\left [{ {\begin{array}{lllll} {\theta _{1} (c_{1})}, {\theta _{1} (c_{2})}, {\theta _{1} (c_{3})\ldots.}, {\theta _{1} (c_{n})} \\ {\theta _{3} (c_{1})}, {\theta _{3} (2_{1})}, {\theta _{13} (c_{3}).\ldots.}, {\theta _{3} (c_{n})} \\ {\theta _{2} (c_{1})}, {\theta _{2} (c_{2})}, {\theta _{2} (c_{3}).\ldots.}, {\theta _{2} (c_{n})} \\ \ldots \\ {\theta _{0} (c_{1})}, {\theta _{0} (c_{2})}, {\theta _{0} (c_{3}).\ldots.}, {\theta _{0} (c_{n})} \\ \end{array}} }\right]}\tag{17}\end{align*}

Deep Convolutional Neural Network (DCNN) with convolutional block is stated as $N_{n}^{c} $ and with ‘n’ filter it is $N_{n}^{f} $.

\begin{align*} P_{ODV}=&\{[{\mathrm {N}}_{f} {\mathrm {S}}_{f} {\mathrm {B}}_{n} {\mathrm {PDA}}]_{I=1}^{N_{n}^{c}},\quad [{\mathrm {N}}_{f} {\mathrm {B}}_{n} {\mathrm {DA}}]_{i=1}^{N_{n}^{f}},{\mathrm {O}}\} \\ \tag{18}\\ \text{Code length}~ c_{l}=&N_{n}^{c} \ast l_{c} +N_{n}^{f} \ast l_{f}\tag{19}\end{align*}

The selection is based on the highest fitness value obtained by each individual. Only those with higher fitness ranking guarantees highest fitness value using elitism roulette wheel selection scheme shown in equation (18) and code length using equation (19).

### C. Cross Over Operator

A pair of DCNN $P_{ODV_{i}} $ and $P_{ODV_{j}} $ is selected, thus a point is located randomly to break the DCNN architecture in two segments. Two new DCNN segment is generated by swapping them, that is $P^{\prime }_{ODV_{i}} $ and $P^{\prime }_{ODV_{j}} $ thus the depth is different compared with parents. Let us assume, cross point ‘$k_{i}$’ is chosen within the ‘$cp_{i} $’convolutional blocks $[{\mathrm {N}}_{f} {\mathrm {S}}_{f} {\mathrm {B}}_{n} {\mathrm {PDA}}]_{cp_{i}} $ on the convolutional arm selected ‘$[{\mathrm {N}}_{f} {\mathrm {B}}_{n} {\mathrm {DA}}]_{P_{ODV_{i}}} $ position is stated as ‘$(cp_{i} -1){}^ \ast l_{c} +x$’ similarly, other convolutional arm $cp_{j}$ and its position is stated as $(cp_{j} -1)^{}\ast l_{c} +x$. The code length of the cross operator is given by the equation (20) and (21).

\begin{align*} C_{l(i)}^{\prime }=&C_{i} +(cp_{i} -cp_{j})^\ast l_{c} \tag{20}\\ C_{l(j)}^{\prime }=&C_{j} +(cp_{j} -cp_{i})^\ast l_{c}\tag{21}\end{align*}

It is clear that ‘8’ learnable layer is needed if the cross point is ‘$k_{i}$’ is positioned at ‘$3l_{c} +1$’ and 11 learnable layers are required at the cross point ‘$k_{j} $’ positioned at ‘$5l_{c} +1$’. Furthermore, after crossover operation the number of layers for DCNN required is 9 and 10 respectively, which is shown in equation (22) and (23),

\begin{align*}&N_{n}^{c} \ast l_{c} +(cp_{i} -1)^\ast 1_{c} +x \tag{22}\\&N_{n}^{c} \ast l_{c} +(cp_{j} -1)^\ast 1_{c} +x\tag{23}\end{align*}

### D. Mutation

DCNN architecture is altered by applying the mutation operator, these mutation operation maintains the diversity in each generation. The new DCNN architecture is accelerated using ‘$q_{m} $’ for the population ‘$P_{ODV} $’ in the range $\left[{ {\frac {8}{L_{n}},0.5} }\right]$. Once the mutation process completed, there will be a change in convolutional block (example from $5\times 5$ to $3\times3$, or $7\times 7$ to $5\times5$), in some case the max pooling layer may be removed and in fifth convolutional layer there will be change in batch normalization process such as 327 to 513. Furthermore, optimizer change from Nadam to RMSprop. The figure 2 shows the proposed GDCNN designer, it tries to improve the individual population with a permitted encoding scheme, CXR image samples are initialized and the fitness of an individual is validated based on the encoding using unique classification problem. On the basis, of present generation new generation is produced using genetic operation which include selection, crossover and the process of mutation. Thus, overall fitness is improved furthermore evolution is performed at generation, until it reaches the defined number.

**FIGURE 1.**
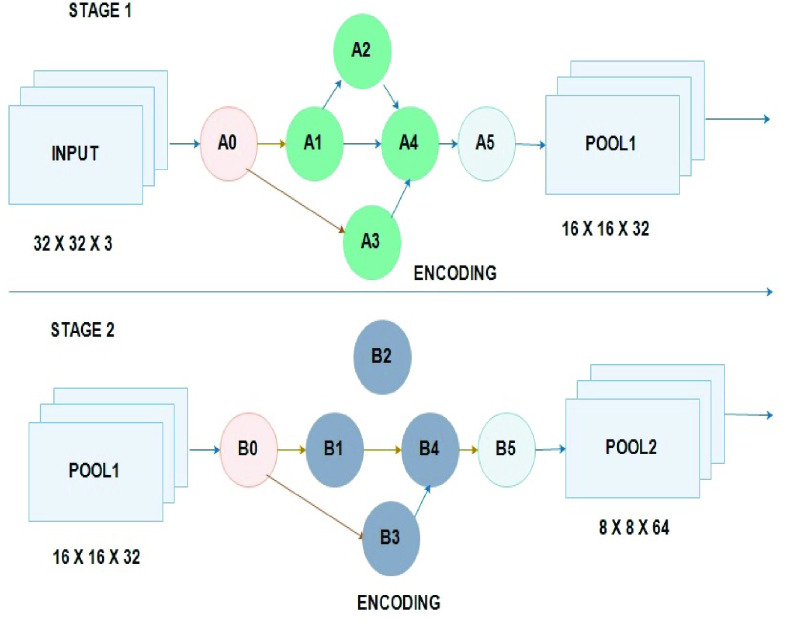
Encoding scheme.

**FIGURE 2.**
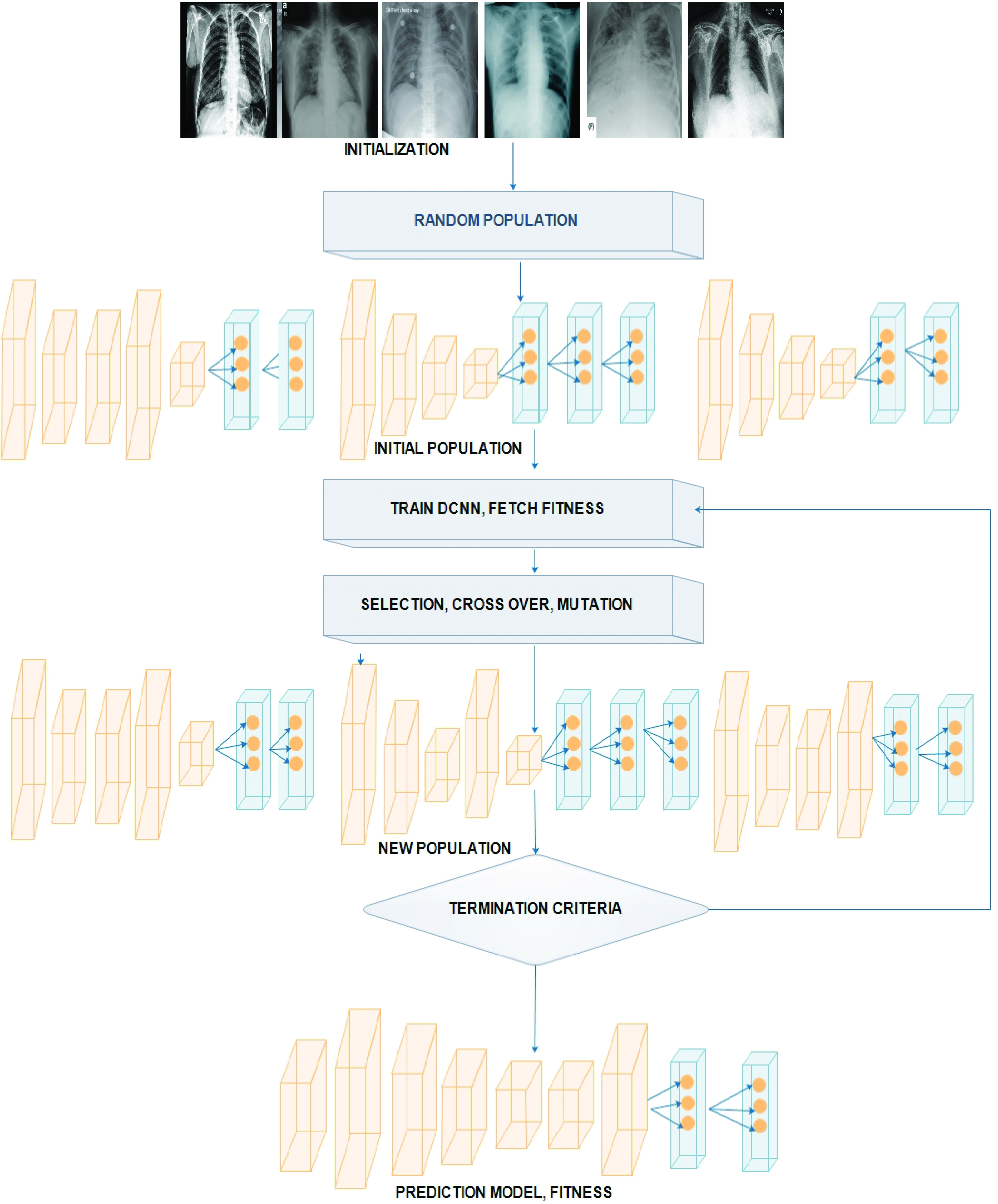
Proposed GDCNN design.

### E. Genetic Deep Convolutional Neural Network_Algorithm

The process involved in the genetic DCNN design architecture is, population initialization where the population is initialized randomly, thereby, it continuously progresses the population on the basis of generation-by-generation for developing better architectures using redefined genetic operations. The Selection operation involves creating a random operation and batch normalization process is performed.

Feeding population to convolution neural network activation is processed and maxpooling is performed, train the GDCNN model for achieving fitness value. Furthermore, model fitness is produced using a generator. Selection, Crossover, and mutation activation are performed. The proposed approach is evaluated on two image classification data set for identifying pneumonia, COVID-19, normal and other pneumonia diseases. Our results show that proposed genetic-based DCNN architecture outperforms well and its performance is comparable to the state of the art.

## IV. Experimental Analysis

The experiment is performed using Intel i7 2.50 GHz and NVIDIA Tesla TitanXp GPU, 512 GB memory, 240 SSD, and tensor flow. Furthermore, the dataset is downloaded from github repository consists of more than 5000 chest x-ray images which include COVID-19, normal, and other pneumonia diseases.

### A. Dataset

The dataset is collected from various parts of the world based on the publications containing chest x-ray images, thus, it requires proper care to verify the labels with board-certified radiologist specialists. The dataset consists of chest x-ray samples of clear sign of COVID-19 using radiologists, and hence these samples contain only anterior-posterior images. Furthermore, this dataset consists of 2,031 chest x-ray training images and 3,040 chest x-ray testing images. The dataset consists of more than 5000 image samples and it is downloaded from the publicly available repository, GitHub link “https://github.com/shervinmin/DeepCovid/tree/master/data” the image source of the dataset is created from the existing dataset such as chexPert dataset consists of non-COVID samples and COVID-chest x-ray-dataset of COVID-19 chest x-ray samples. The dataset contains the parameters such as age and sex, thus 102 COVID-19 chest x-ray image with a clear indication of COVID-19 certified by a specialist in radiology. Thus out of 5000 images, 71 chest x-ray images are not taken into account as they are less-reliable posterior-anterior images with COVID-19. Data augmentation is used for increasing the sample size and thus after applying rotation, flipping, over-sampling and small distortion, 496 COVID-19 image samples from the training set. To increase the non-COVID image samples additional images are fetched from the ChexPer dataset, as it’s a large publicly available chest x-ray dataset. This dataset consists of 2,24,316 chest x-ray images of 65,240 patients, furthermore, it is labeled based on 14 sub-categories such as edema, no-finding, bacterial pneumonia, Acute respiratory distress syndrome (ARDS), COVID-19, influenza, fungal pneumonia, klebsiella, Middle East respiratory syndrome (MERS), legionella, mycoplasma, lipoid, pneumocystis, viral pneumonia, pneumonia, streptococcus, and SARS. Training set 496 augmented images for COVID-19 and non-COVID 2000 images, the test set 40 for COVID-19 and non-COVID 3000 images are used.

Algorithm 1 **Genetic Deep Learning Convolution Neural Network_Algorithm**
Input: *5000 chest x-ray images (collection of images, training and test data).*
Output: *Accuracy, sample loss, val _loss,val_acc.*
Step1: *Initialization Input the 5000 chest x-ray images (training and test data)*
Step2: *Create random operation*
*Batch Normalization process*
Step3: *Feed population to Convolution neural network Activation,*
  *conv2D(512, (3 $x$ 3), padding = same, usebias = false)*
  *maxpooling (pool size = (3, 3)*
  *Dropout*
Step4: *Train GDCNN and get its fitness*
  *modelfit:generator(datagen.flow(x train, y train, batch size = batch = size),*
  *steps per epoch = x train,shape[0] (batch size, epoch = epochs,validation data = (x test, y test),*
  *callbacks = [plot])*
  *else*
  *modelfit:(x train, y train, batch size = batch size, epoch = epochs, validation data = (x test, y test), shuffle = true, callbacks = [plot]*
Step 5: *selection, crossover, mutation*
  *Activation,*
  *conv2D (512, ($3\times 3$), padding = same, use bias = false)*
  *maxpooling (pool size = (3, 3)*
  *Dropout*
Step 6: *New populations train GDCNN and get its fitness*
  *Evaluate solution based on fitness value*
Step 7: *Check optimal solution based of fitness function*
  *If (optimal solution == fitness value)*
  *Optimal solution obtained*
Step 8: *Fitness value (optimal solution)*
  *Return optimal solution*

Some of the disease cases selected are pulmonary edema 293, pleural effusion 311, Chronic Obstructive Pulmonary Disease (COPD) of 315 images, and Pulmonary Fibrosis of 280 images.

### B. Data Limitations

The dataset contains only small samples of COVID-19 infected cases, hence patients with severe symptoms also need to be analyzed. Furthermore, cases with mild symptoms missing, and some people are even quarantined without examining them. Some of the pneumonia samples are collected previously and there is no suspected of coronavirus infection, finally, data deals to demographic characteristics and other risk factor related to patients is not available.

### C. Accuracy

Accuracy is one of the important metrics used for evaluating the classification models, accuracy states whether our model is right and it is defined as the number of correct predictions of COVID-19 to the total number of prediction samples. The confidence interval of the accuracy rates can be calculated as equation 24,

\begin{equation*} r=z\sqrt {\frac {accuracy(1-{\mathrm {accuracy}})}{N} }\tag{24}\end{equation*}

Accuracy is also stated as the sum of True Positive (TP) and True Negative (TN) to the sum of True Positive (TP), True Negative (TN), False Positive (FP) and False Negative (FN) using equation (25), as shown at the bottom of the page.

**Figure:** \begin{equation*} Accuracy=\frac {\sum \limits _{c} {(TruePositive({\mathrm {TP}}))+TrueNegative({\mathrm {TN}}))}}{\sum \limits _{c} (TruePositive({\mathrm {TP}})+{\mathrm {TrueNegative(TN)+FalsePositive(FP)+FalseNegative(FN)}})}\tag{25}\end{equation*}

### D. Sensitivity

Sensitivity and specificity are the important benchmark metric for evaluation of classification and thus sensitivity states True Positive (TP) to the sum of True Positive (TP) and False Negative (FN). Hence, it is given by equation (26)

\begin{align*} {Sensitivity}=\frac {\sum _{c}({TruePositive}(\mathrm {TP}))}{\sum _{c}({TruePositive}(\mathrm {TP})+{FalseNegative}(\mathrm {FN}))} \\\tag{26}\end{align*}

### E. Specificity

Specificity is defined as the True Positive (TP) to the sum of True Positive (TP) and False Positive (FP). Calculated using equation (27),

\begin{align*} {Specificity}=\frac {\sum _{c}({TruePositive}(\mathrm {TP}))}{\sum _{C}({TruePositive}(\mathrm {TP})+{FalsePositive}(\mathrm {FP}))} \\\tag{27}\end{align*}

### F. Precision

Precision is calculated as the sum of True Positive of all the classes to the summation of all classes True Positive (TP) to the False Positive (FP), it is given by equation (28)

\begin{equation*} Precision=\frac {\sum \limits _{c} {\big (TruePositive \big)_{c}}}{\sum \limits _{c} {\big(TruePositive} \big)_{c} +\sum \limits _{c} {\big(FalsePositive} \big)}\tag{28}\end{equation*}

### G. Recall

Recall is measured as the summation of all class True Positive (TP) to the summation of class True Positive (TP) and False Negative (FN), it is stated by the equation (17)

\begin{equation*} {\mathrm {Re}}call =\frac {\sum \limits _{c}({TruePositive})_{c}}{\sum \limits _{c}({TruePositive})_{c}+\sum \limits _{c}({FalseNegative})}\tag{29}\end{equation*}

### H. F1-Score

F1-score is used to measure the balance between the precision and recall. Furthermore, it is stated as the twice the product of precision and recall to the sum of product and sensitivity. Equation (30) states the precision calculation,

\begin{equation*} F1\_{}score=2\left({\frac {precision\ast recall}{precision+recall}}\right)\tag{30}\end{equation*}

F1_score is stated as the weighted average of precision and recall, ‘1’ is stated as the best score of ‘F1’ and ‘0’ as the worst score.

### I. Log Loss

Log loss state the logarithmic loss function and it is stated by equation (31)

\begin{equation*} \log loss=\frac {-1}{N}\sum \limits _{i=1}^{N} {(y_{i} (\log (p)_{i})+(1-y_{i})\log (1-p_{i}))}\tag{31}\end{equation*}

Four cases of log loss are,

Case 1: \begin{equation*} y_{i} =1,\quad p_{i} =high, 1-y_{i} =0, 1-p_{i} =low\tag{32}\end{equation*}
  When $y_{i} =1, $ and $p_{i} =high$, it states that the model is working perfectly, this is generally due to the true value of response mostly agreed with highest probability. There is a “n” expand in sum as $y_{i} (\log (p)_{i})$ is high and the other term is zero as
  \begin{equation*} 1-y_{i} =1-1=0\tag{33}\end{equation*}
  Thus higher the value there is the possible influence in sum and in mean, this is mainly due to
  \begin{align*} p_{i}>&p_{i} -1 \tag{34}\\ \log (p_{i})>&\log (p_{i} -1)\tag{35}\end{align*}
Case 2: \begin{equation*} y_{i} =1,\quad p_{i} =low, 1-y_{i} =0, 1-p_{i} =high\tag{36}\end{equation*}
  In this case $y_{i} =1$ and $p_{i} =low$ it is totally adverse as the probability of y is 1 and being low furthermore, as the value of $y=1$ there is little impact on sum.
Case 3: \begin{equation*} y_{i} =0,\quad p_{i} =low, 1-y_{i} =0, 1-p_{i} =high\tag{37}\end{equation*}
Case 4: \begin{equation*} y_{i} =0,\quad p_{i} =high, 1-y_{i} =0, 1-p_{i} =low\tag{38}\end{equation*}
  In case 3 and case 4 there is as drastically expand in sum and thus it affects the model.

### J. Confusion Matrix

**FIGURE 3.**
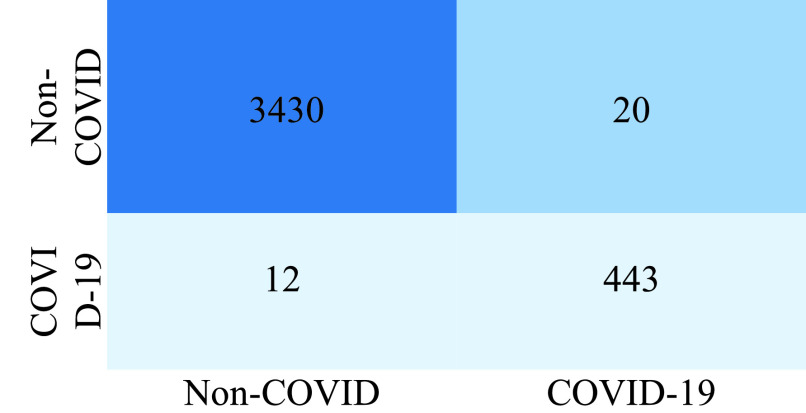
Confusion matrix of COVID-19.

Confusion matrix gives the overall performance of the model and the output is given in the form of a matrix. It is clear from the confusion matrix in figure 3, that a normal person is 3430 these are the person, who are not affected by COVID-19. 20 of the persons are likelihood of having COVID-19, 12 people may have COVID-19 and 443 persons are confirmed COVID-19.

**FIGURE 4.**
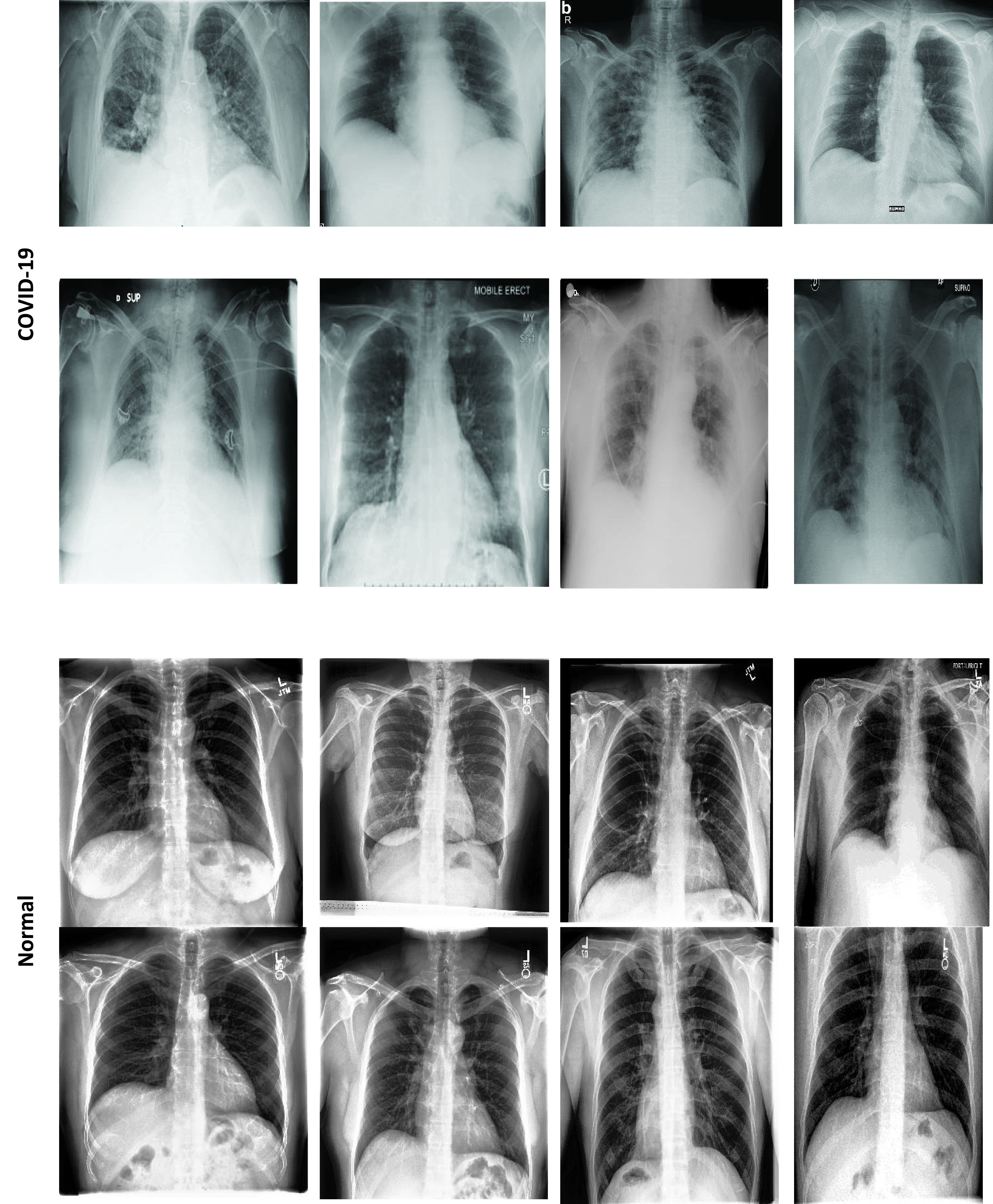
COVID-19 and normal chest X-Ray images [64].

**FIGURE 5.**
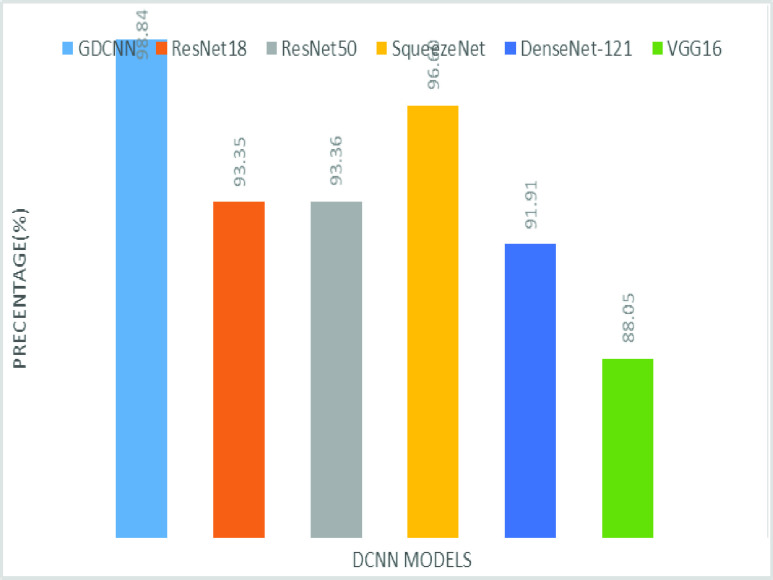
Comparison of accuracy.

**FIGURE 6.**
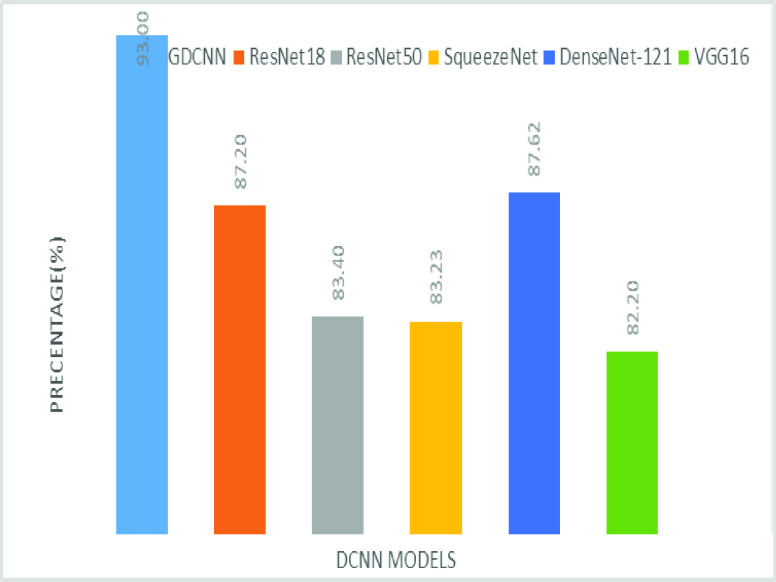
Comparison of precision.

**FIGURE 7.**
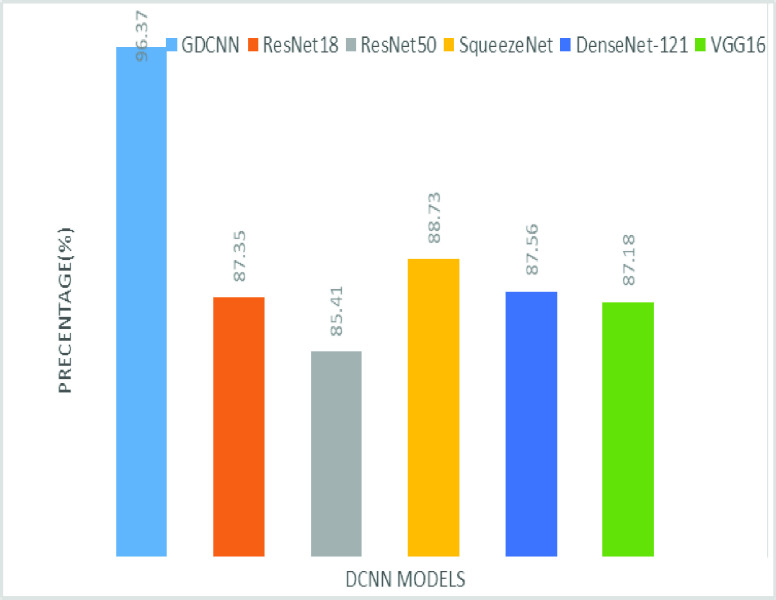
Comparison of F1-score.

The validation of the proposed model is evaluated using some of the benchmark metric functions such as accuracy, precision, sensitivity, specificity, and F1-score. The experiment is carried out for 100 trail to achieve better performance. Figure 4 shows the chest x-ray image sample of COVID-19 and healthy lung persons, persons who are affected by COVID-19 CXR image are not clearly visible whereas, normal persons’ lungs images are clearly identifiable. Table 3 shows the confusion matrix for pneumonia and from the table, it is clear that the number of people affected by COVID-19, normal and other pneumonia is easily identifiable. Table 4 shows the performance metric for evaluation of the proposed model with other existing models and Table 5 shows the analysis of accuracy with proposed and other existing models for 10 trails with 100 iterations. Figure 5 shows the performance of accuracy and it is compared with the proposed model with the other existing models such as ResNet18, ResNet50, SqueezeNet, Densenet-121, and VGG16. The proposed model has the highest accuracy of 98.84%, whereas, VGG16 has the lowest accuracy of 88.05%. The other models like ResNet18, ResNet50, and Densenet-121 with an accuracy of 92.7% furthermore, SqueezeNet with 96.60% accuracy is comparable with the proposed model. The accuracy bar graph shows that the proposed model outperforms well than the rest of the existing models. Figure 6 shows the comparative analysis of proposed model precision along with the other existing models, GDCNN model with the highest precision of 93.0%, whereas, ResNet18 and DenseNet121 with a precision of 87%. The other three models like ResNet50, SqueezeNet, and VGG16 with an average precision of 82.13%. The precision bar graph shows that other existing models have an average precision of 85.34% and thus proposed a model with higher precision of 93.0%. Figure 7 shows the analysis of the F1-score bar graph with the other models, thus, the proposed model with an F1-score of 96.37%, whereas other models with an average F1-score of 86.53%. Thus the F1-score shows how the model best in terms of prediction of COVID-19 and normal diseases. The proposed model F1-score indicates that better classifications are performed.

**TABLE 3:** Confusion Matrix for Pneumonia

Sl.No.	Disease Name	COVID-19	Edema	Effusion	Emphys	Fibrosis	Pneumonia	Normal
1	COVID-19	443	1	4	4	7	3	1
2	Edema	1	232	36	34	11	0	0
3	Effusion	2	31	161	58	37	0	0
4	Emphys	3	12	54	156	40	0	0
5	Fibrosis	3	17	56	63	184	0	0
6	Pneumonia	1	0	0	0	1	907	0
7	Normal	2	0	0	0	0	0	1340

**TABLE 4:** Performance Metric

Sl.No.	Models	Accuracy	Precision	Recall	F1-score	Sensitivity	Specificity
1	GDCNN	98.84	93.00	100.00	96.37	100.00	97.00
2	ResNet18	93.35	87.20	87.50	87.35	87.50	99.20
3	ResNet50	93.36	83.40	87.51	85.41	87.51	99.21
4	SqueezeNet	96.60	83.23	95.00	88.73	95.00	98.20
5	DenseNet-121	91.91	87.62	87.50	87.56	87.50	96.32
6	VGG16	88.05	82.20	92.80	87.18	92.80	83.30

**TABLE 5:** Analysis of Accuracy for 10 Trail

Sl.No.	GDCNN	ResNet18	ResNet50	SqueezeNet	DenseNet-121	VGG16
1	95.83	89.03	89.52	92.21	86.97	84.12
2	96.35	89.26	89.84	92.4	87.4	85.04
3	96.45	90.03	89.88	92.77	87.79	85.16
4	96.59	90.04	90.63	92.92	88.16	85.16
5	97.90	90.35	91.64	93.54	88.32	85.40
6	97.93	91.19	91.68	94.31	88.41	85.54
7	97.94	91.40	91.99	95.25	88.45	86.17
8	98.04	91.72	92.83	95.49	89.03	86.27
9	98.55	92.08	92.92	95.56	89.93	87.35
10	98.84	92.31	93.71	96.6	91.62	88.05

**FIGURE 8.**
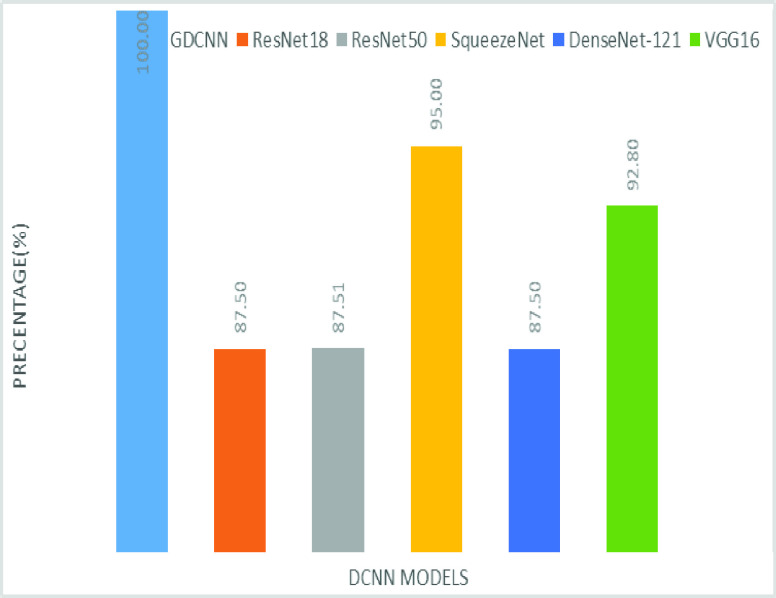
Comparison of sensitivity.

**FIGURE 9.**
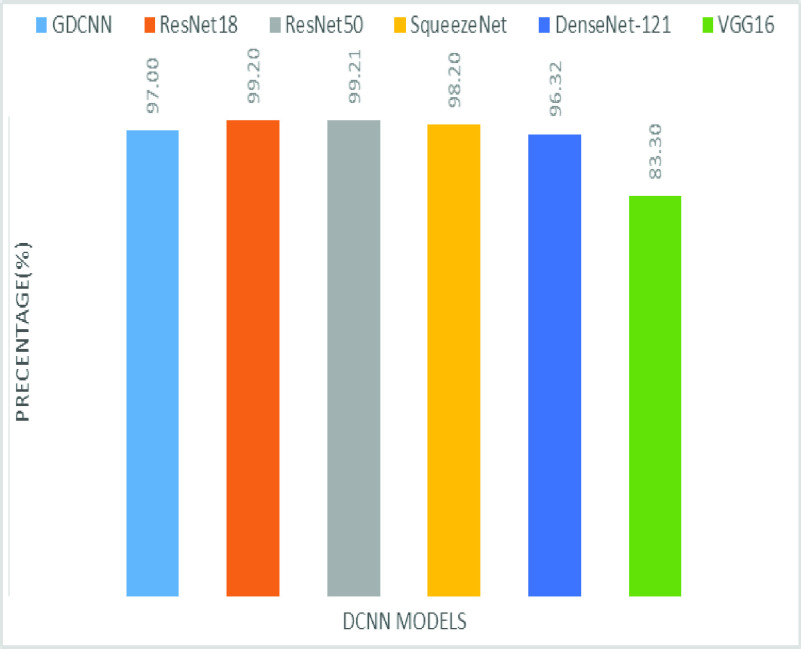
Comparison of specificity.

**FIGURE 10.**
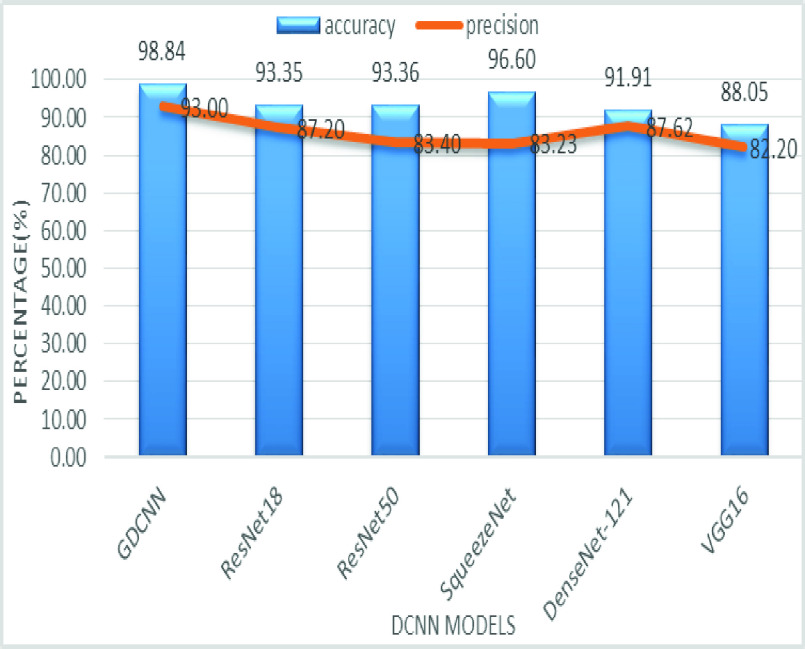
Comparison of accuracy and precision.

Figure 8 shows the sensitivity bar graph analysis of proposed models with other available models, 100% sensitivity is achieved for the proposed model, whereas, ResNet18, ResNet50, and Densenet121 with an average of 87.50%. Squeezenet and VGG16 with a sensitivity of 93.53% are achieved. Sensitivity is one of the important metrics to validate the performance of the proposed model. Figure 9 shows the specificity bar graph analysis in terms of the proposed model with other existing models, specificity is one of the significant benchmark metrics for performance evaluation. Specificity with an average of 97% is achieved for GDCNN, ResNet18, ResNet50, Squeezenet, and Densenet-121. Minimum specificity of 83.30% is achieved for VGG16. Figure 10 shows the combo chart of accuracy and precision analysis of the proposed models with existing models. The combined analysis of both accuracy and precision is plotted in the graph, accuracy is represented in terms of a bar graph and precision is shown in the form of a line graph. The proposed model with an accuracy of 98.84% and a precision of 93.0% is achieved thus both of them show higher performance than the existing model. Meanwhile, Squeezenet with an accuracy of 96.60%, whereas, the precision of only 83.23% is achieved. VGG16 with a minimum accuracy of 88.05 and a precision of 82.20 is obtained. The combo graph states that both accuracy and precision with higher performance is obtained.

**FIGURE 11.**
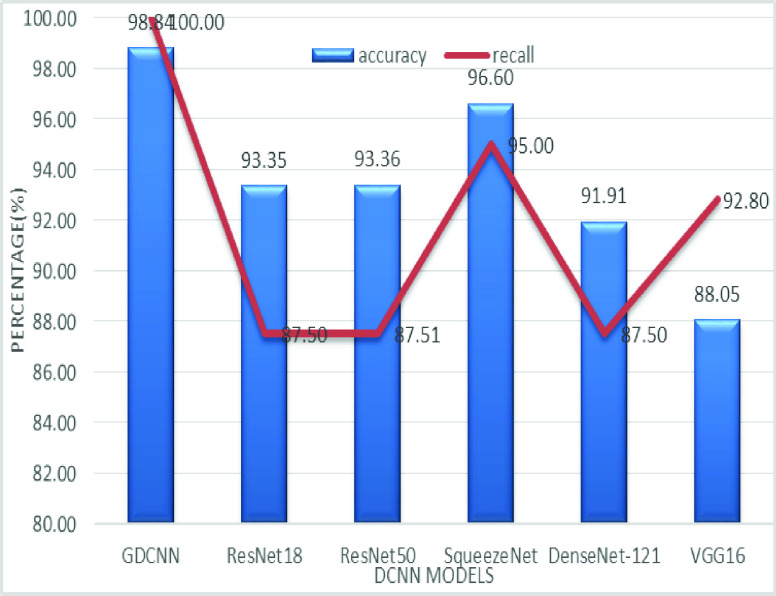
Comparison of accuracy and recall.

Figure 11 shows the accuracy and recall performance analysis, it is clear that the GDCNN model with an accuracy of 98.84% and 100% recall is achieved able using the proposed model, on the other hand, Squeezenet with 96.60% and recall of 95% is obtained. Resnet18, Resnet50, and Densenet with an average recall of 87.5% are achievable, whereas, the accuracy of 92.36% is obtained and VGG16 with the lowest recall rate of 88.05% and accuracy of 92.80 is obtained. It is the same for figure 13 as both recall and sensitivity are the same.

**FIGURE 12.**
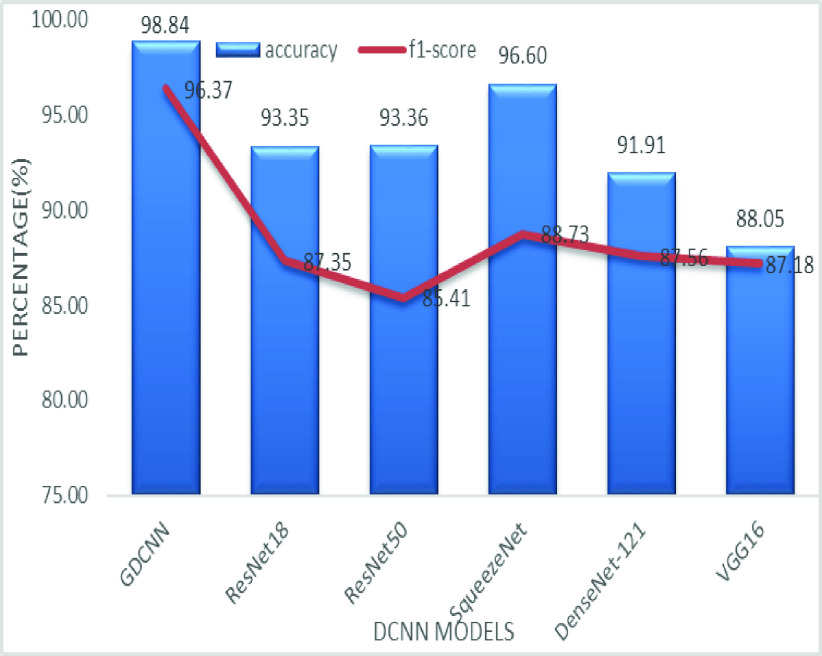
Comparison of accuracy and F1-score.

**FIGURE 13.**
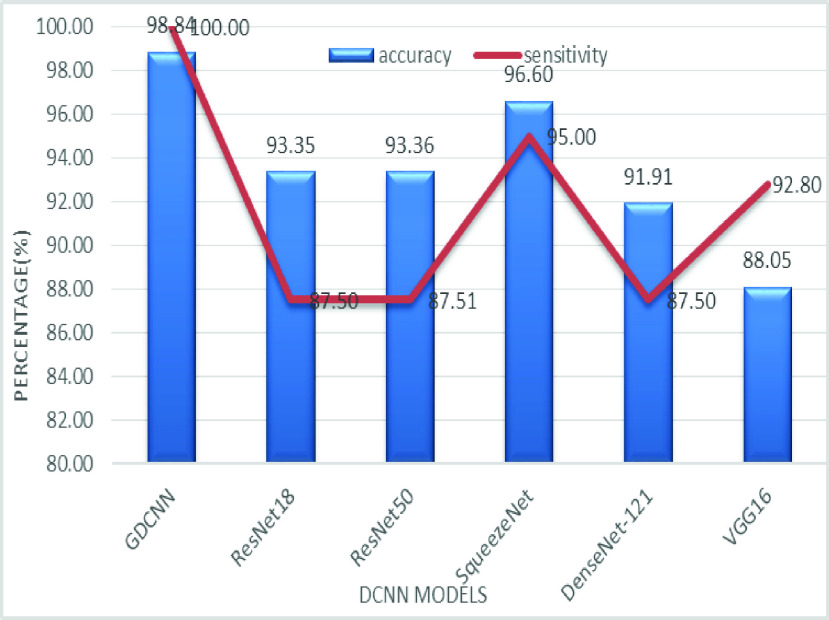
Comparison of accuracy and sensitivity.

Figure 12 shows the comparative analysis of accuracy with the F1-score, for the proposed GDCNN model with an accuracy of 98.84% and an F1-score of 96.37% is achieved, whereas for the rest of the models it is comparatively low. F1-score is drastically lower than compared with the accuracy for the other existing models and VGG F1-score of 87.18 is obtained.

**FIGURE 14.**
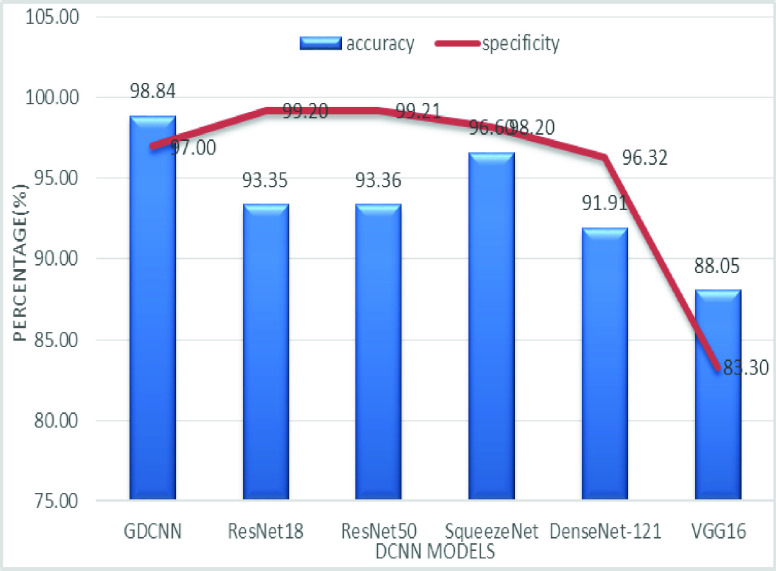
Comparison of accuracy and specificity.

**FIGURE 15.**
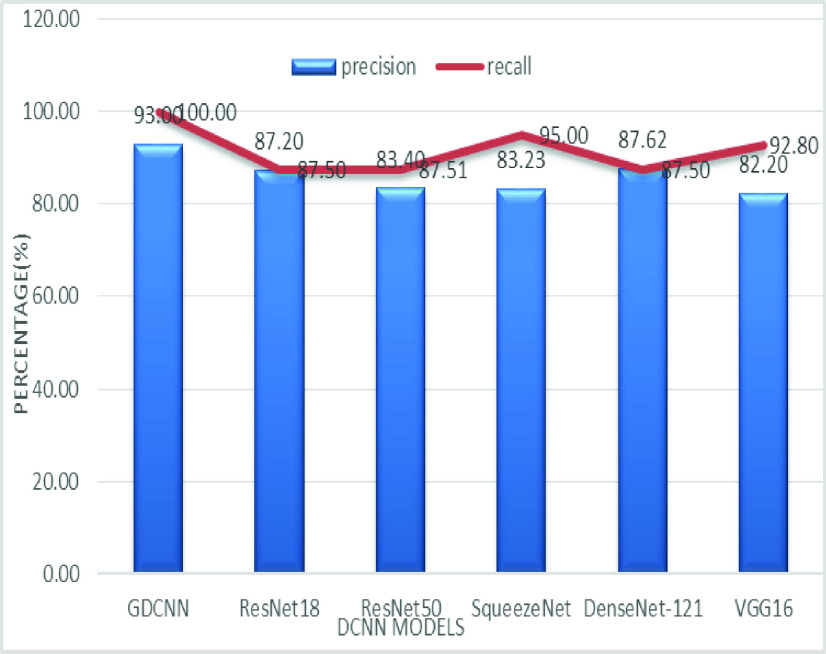
Comparison of precision and recall.

**FIGURE 16.**
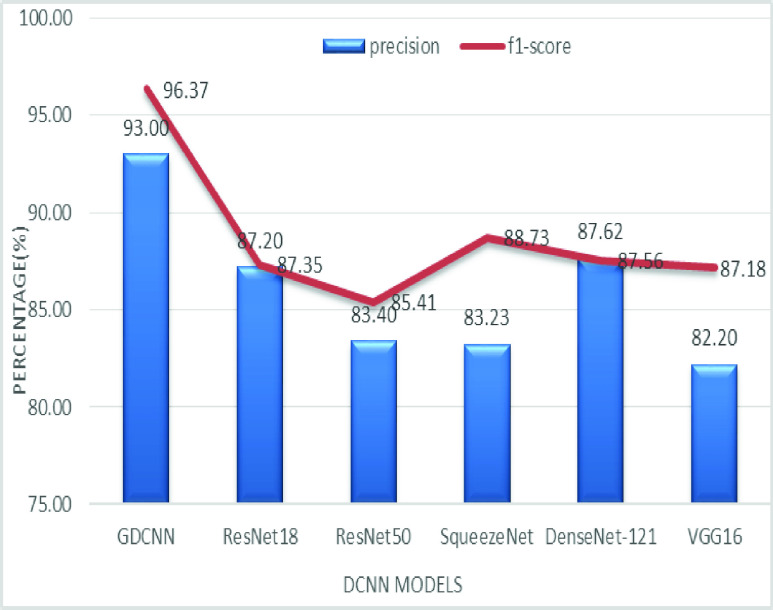
Comparison of precision and F1-score.

**FIGURE 17.**
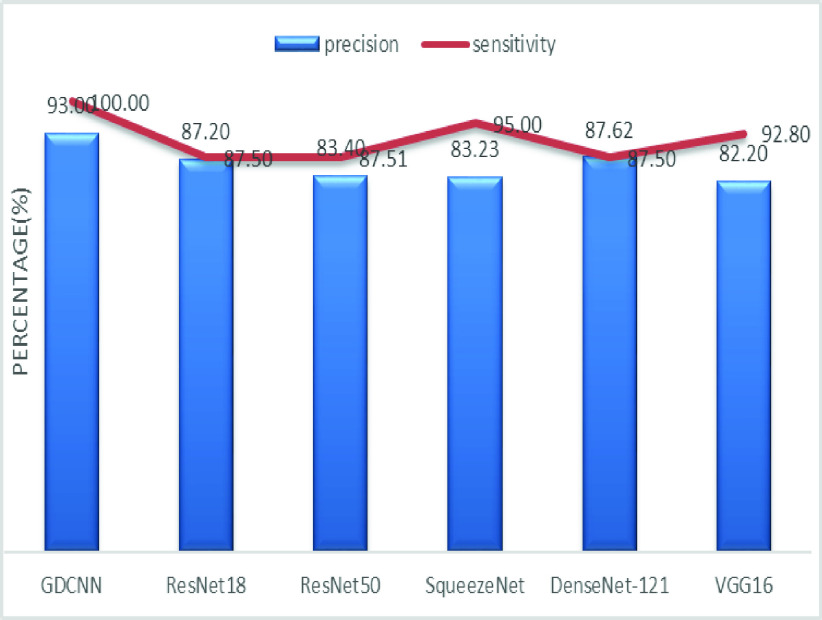
Comparison of precision and sensitivity.

Figure 14 shows the comparative analysis of accuracy and specificity analysis of the proposed model with other models, furthermore, it is clear that both accuracy and specificity are comparatively high for both proposed as well as other existing models. Figure 15 shows the comparison of bar graph between precision and recall for various models. It is clear from the figure that the proposed GDCNN model performs well compared to other existing models such as ResNet18, ResNet50, SqueezeNet, DenseNet-121 and VGG16. Proposed GDCNN models with 93.00% of precision and recall of 100% is achieved, whereas ResNet18, DenseNet-121 with precision of 87% is achieved. Recall value of ResNet18, SqueezeNet and VGG16 is above 92%. The minimum value of precision and recall is for ResNet50 with 83.40% and 87.51% considerably. Figure 16 represents a comparison bar for precision and f1-score, from above comparison, it states that proposed GDCNN is considerably high with 93% of precision and 96.37% of f1-score. Lowest precision and f1-score is obtained for ResNet50 and SqueezeNet, with precision of 83.40 and recall of 85.41% for ResNet50 and for SqueezeNet it is 83.23% and 88.73%. Figure 17 shows the comparison graph for precision and sensitivity for various models. Proposed GDCNN model with precision of 93.00% and a sensitivity of 100.00% is achieved, whereas reset of the models is low. ResNet18, ResNet50 and DenseNet-121 precision value is 87.5% and there sensitivity values are 87.20%, 83.40 and 87.62% respectively.

**FIGURE 18.**
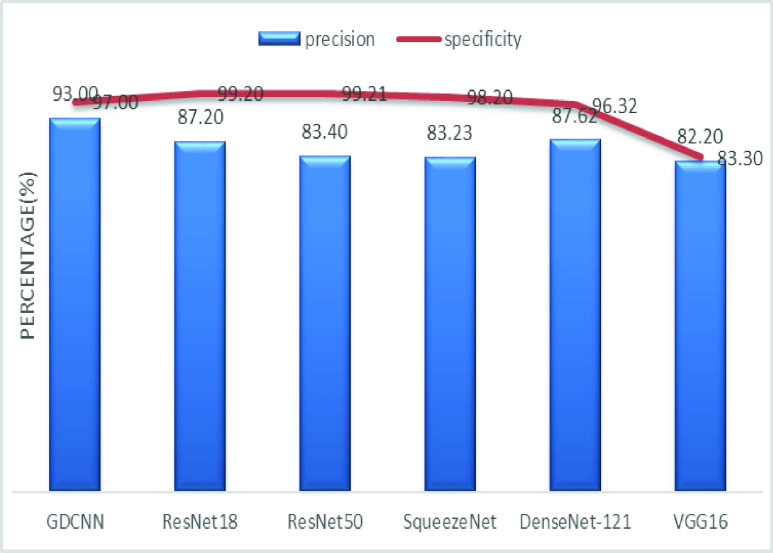
Comparison of precision and specificity.

**FIGURE 19.**
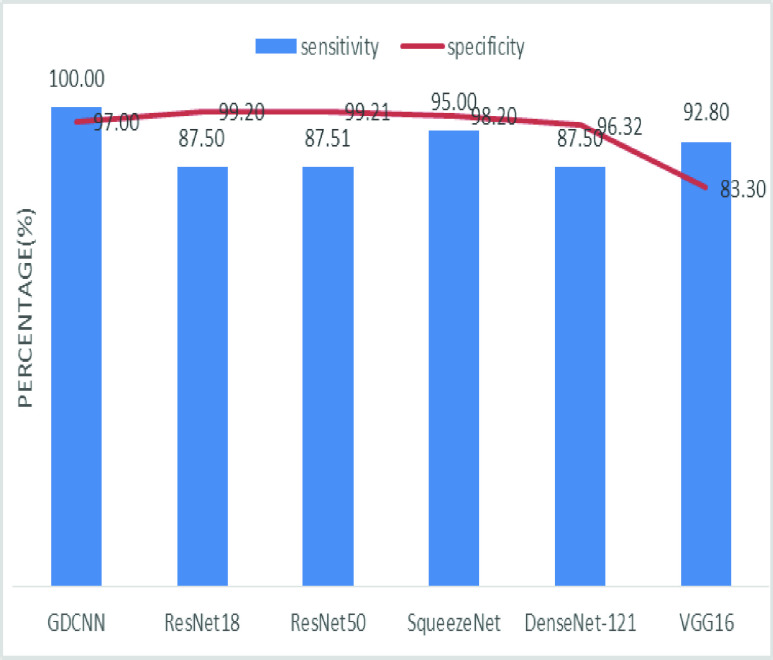
Comparison of sensitivity and specificity.

Figure 18 depicts the precision and specificity comparison graph, proposed GDCNN with 93.00% and 97.00% obviously. All the models try to perform more or less same, However, VGG16 performs lowest of all the models with precision of 83.30% and specificity of 82.20%. Figure 19 shows the sensitivity and specificity comparable graph for proposed models and it is compared with the other existing models. Sensitivity and specificity out perform well with 100.00% and 97.00% considerably. All the other models also try to perform, but it lacks in sensitivity as it’s approximately 87.50 for ResNet18, ResNet50, SqueezeNet and DenseNet-121 whereas specificity above 90% is achieved.

**FIGURE 20.**
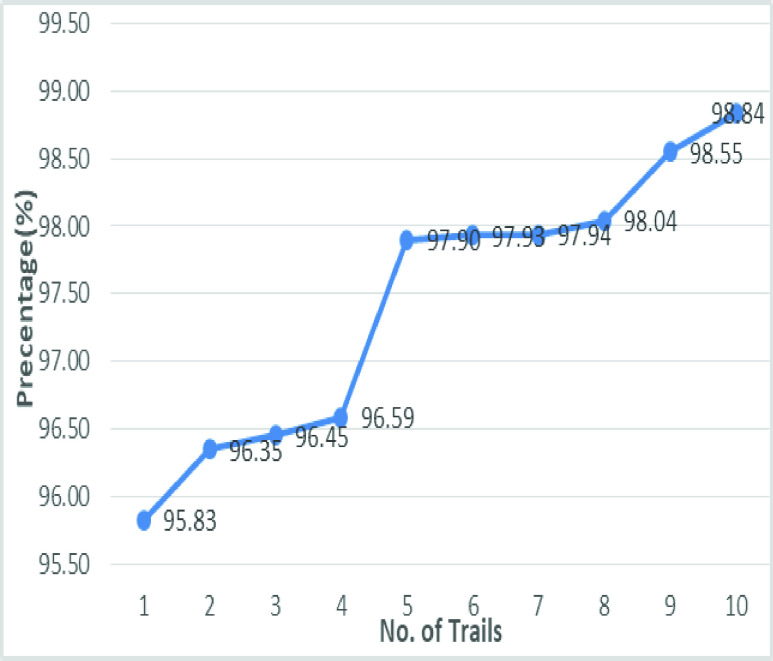
Analysis of accuracy for GDCNN.

**FIGURE 21.**
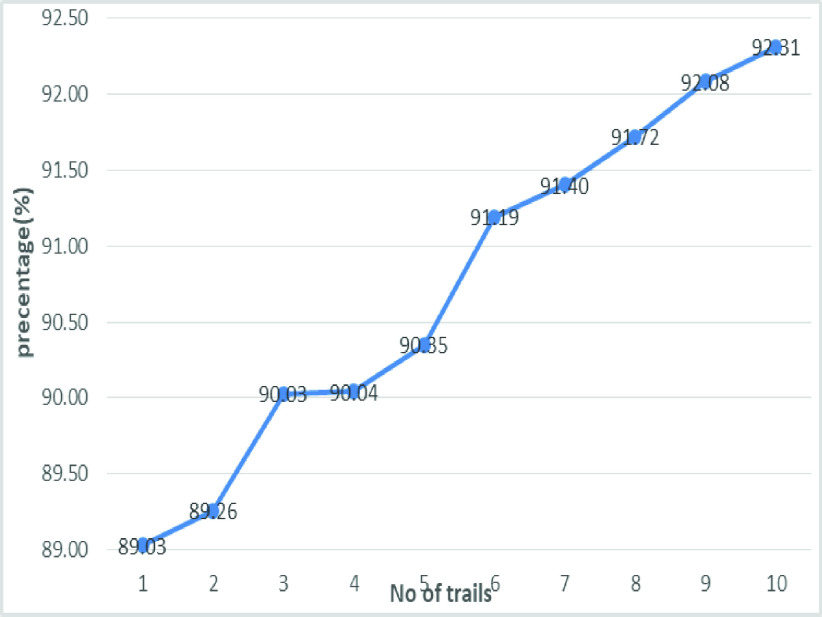
Analysis of accuracy for Resnet18.

**FIGURE 22.**
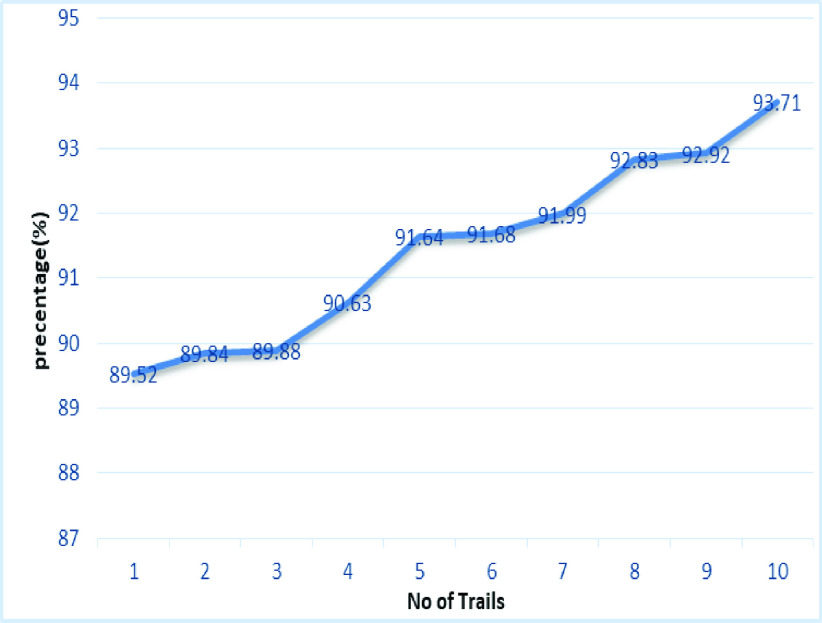
Analysis of accuracy for Resnet50.

Figure 20 shows the line graph for the proposed GDCNN models with 100 iterations for 10 trails. Accuracy is predicted for various trails starting 95.83% to a high of 98.84%, this is mainly due to training models as the models get trained better accuracy is achieved. Figure 21 and 22 represent the line graph for ResNet18 and ResNet50. Initially the accuracy for both the model is 89% and ResNet18 better accuracy of 92.81 is achieved, whereas, for ResNet50 is 93.71% is achieved for 10 trails.

**FIGURE 23.**
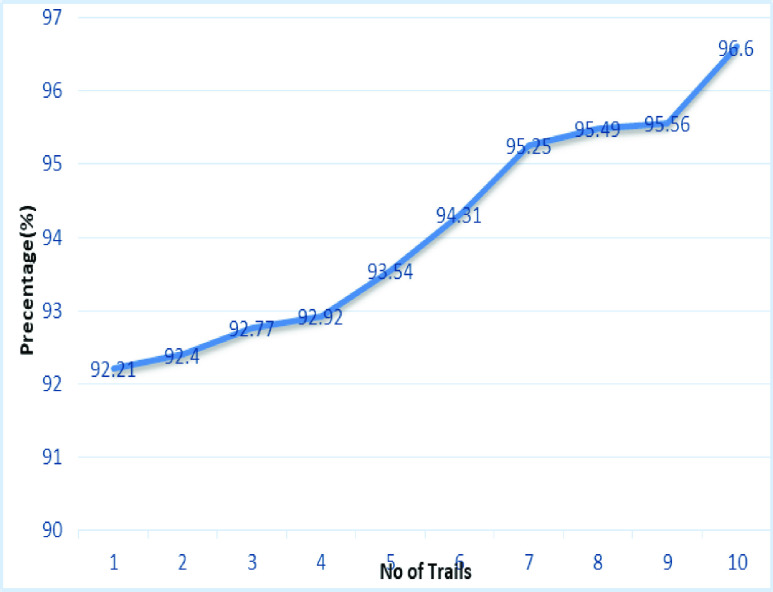
Analysis of accuracy for Squeezenet.

**FIGURE 24.**
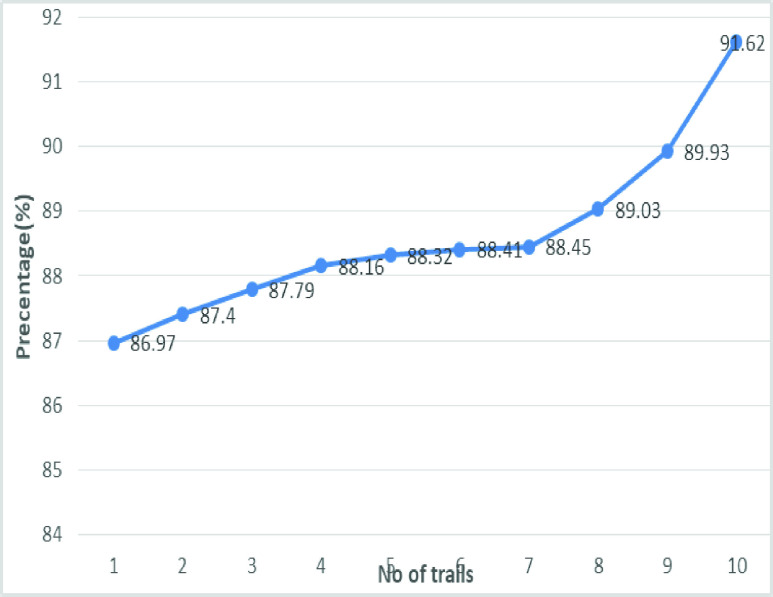
Analysis of accuracy for Densenet121.

**FIGURE 25.**
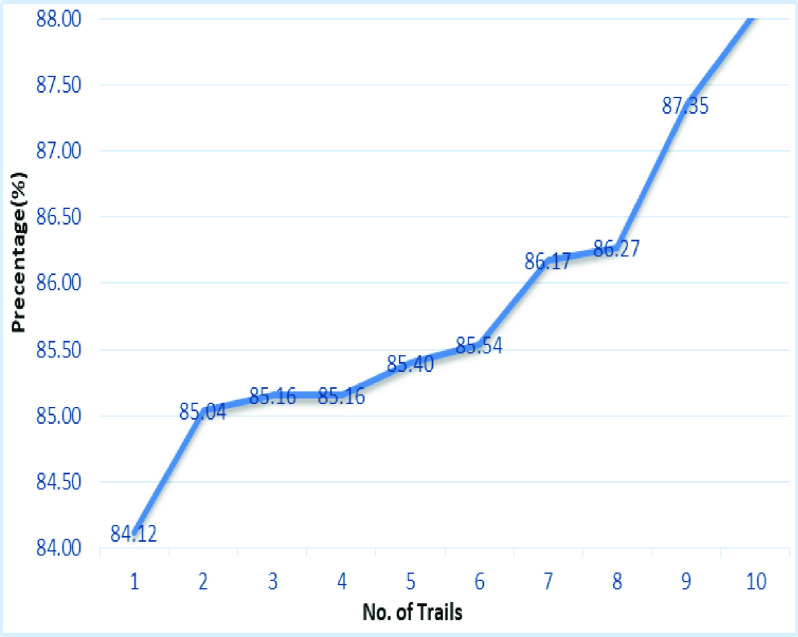
Analysis of accuracy for VGG16.

Figure 22 and 23 shows the accuracy analysis for SqueezeNet and DenseNet-121 with 100 iterations for 10 trails. For SqueezeNet maximum of 96.6% of accuracy is achieved, whereas for DenseNet-121 it is 94.62%. A gradual increase in the accuracy is notifiable for DenseNet-121, as its initial accuracy of 86.97% and then achieving up to 94.62%. Figure 24 shows the accuracy analysis for 10 trails with each 100 iterations for VGG16. It is clear that accuracy also tends to increase gradually as the number of trail increases. Figure 25 line graph shows the comparative analysis of accuracy of the proposed model with the available models, thus the proposed model performs well with respect to the number of trails. VGG16 is with the lowest accuracy, while resnet18 and resnet50 both models with similar accuracy is achieved.

**FIGURE 26.**
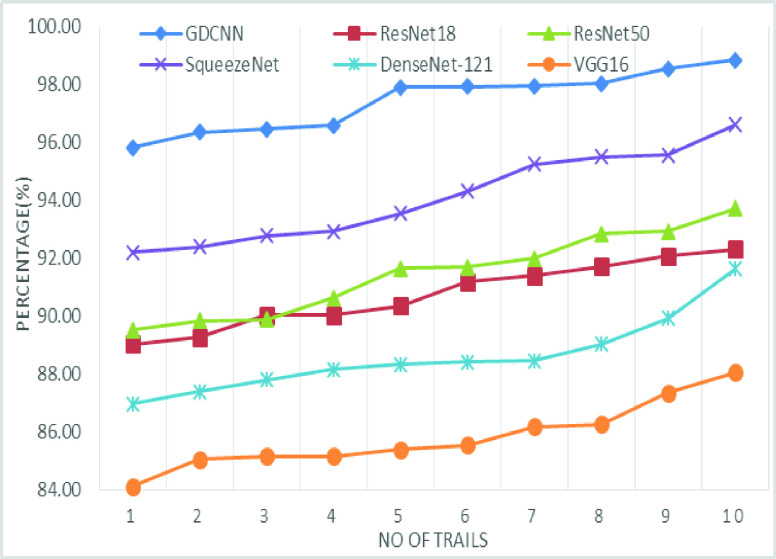
Comparison of accuracy for various models.

Our main goal of this research is to develop GDCNN based approaches for predicting the lung infection due to COVID-19 using chest x-ray images. Healthy versus pneumonia samples are identified with an accuracy of 98.72%

### K. Expected Outcomes

The tool is developed based on the GDCNN model which is very helpful for physicians and act confident in the treatment of a COVID-19 affected patient, while they are waiting for the second opinion confirmation with the radiologist. Furthermore, it provides a measurable score to consider and to use in research studies.

### L. Discussion on Complexity

Training of each chest x-ray image generated by DCNN is fixed to 100 epochs, thus the proposed GDCNN has high computation and space complexity, and this is mainly due to storing and evaluating a huge amount of DCNN structure. Lacks security as the health care data are stored in the cloud environment and hence the proper security mechanism need to be implemented for retrieving data [67], [68].

## V. Conclusion

The fast spread of COVID-19 creates a pandemic all over the world as there exists an exponential increase in the number of cases. Early diagnosis of diseases is in urgent need in the treatment of COVID-19, which should be faster and cheaper. In the above context, a deep learning method is used for the prediction of COVID-19 from CXR image samples. In the real world, only a few people have bee affected by pneumonia whereas, many of them remain unaffected. Hence, there arises an imbalance in the prediction of pneumonia between the affected person and a normal person. In this research, the GDCNNN method is proposed for classifying COVID-19 and normal person, and it is done through CXR image samples. More than 5000 image samples are taken from the publicly available repository, consisting of pneumonia, healthy lung images, and other pneumonia diseases. The proposed method with F1-score of 0.96337, val_accuracy of 0.99 (99.0%), loss of 0.32 and val_loss of 0.05 is achieved. Furthermore, it is compared with other existing models such as resenet18, resenet50, SqueezeNet, Densenet-121, and VGG16 to evaluate the performance of the proposed model. It is clear from the analysis table that the proposed method outperforms well than compared to the existing model. The main aim of the research is to provide a better identification rate for COVID-19 prediction in the earlier stage of diagnosis and provide greater help emergency of patients in earlier treatment. The organization can use this model for earlier prediction COVID-19 as GDCNN tool hosted resides in the cloud computing environment. The health care system can use this tool for earlier diagnosis of diseases. In the future we hope to apply this method for a large scale database for achieving better hierarchical classification accuracy.

## Software Availability

Our prediction model is available online at https://github.com/BABUKARTHIKRG/covid19.git.

Few interactive graphs can be seen at https://collaboration.coraltele.com/covid2/.
